# Polymeric Nanocarrier-Based Drug Formulations for Enhancing Nose-to-Brain Delivery

**DOI:** 10.3390/pharmaceutics17101242

**Published:** 2025-09-23

**Authors:** Tobeka Naki, Sijongesonke Peter, Sibusiso Alven

**Affiliations:** 1 Department of Chemistry, University of Fort Hare, Alice Campus, Alice 5700, Eastern Cape, South Africa; 201414787@ufh.ac.za; 2 Department of Chemistry, Nelson Mandela University, Gqeberha 6001, Eastern Cape, South Africa; s217616712@mandela.ac.za

**Keywords:** nanocarriers, polymers, blood–brain barrier, cancer, HIV, Alzheimer’s disease, Parkinson’s and Huntington’s disease

## Abstract

Neurological-related diseases are among the most debilitating and difficult to manage. Many possible pharmacological treatments for neurological diseases struggle to cross the blood–brain barrier (BBB) to achieve concentrations that can produce a therapeutic benefit. This is primarily because of the existence of the BBB, which poses significant hurdles for both therapeutic and diagnostic efforts by restricting the entry of most medications. Nasal-to-brain drug transportation has surfaced as an encouraging approach to tackle the difficulties linked with conventional drug administration techniques for neurological disorders. In response, innovative methods for improving drug delivery focus on breaking down the BBB via physical techniques, including optical and photothermal therapy, electrical stimulation, and acoustic or mechanical stimulation. Nanocarriers represent a promising approach for facilitating nasal systemic and brain delivery of active compounds. Hence, the achievement of therapeutically relevant concentrations of exogenous molecules within the body is significantly contingent upon the nanocarriers’ capability to surpass biological barriers. Polymers in nanocarrier formulations can result in significantly enhanced nose-to-brain drug delivery by protecting drugs from premature biodegradation, increasing permeability, improving mucoadhesion, and targeting specific cells in the brain. Polymeric nanocarriers are frequently functionalized with cell-penetrating peptides to further improve the specificity of the loaded therapeutic molecules. This review focuses on the use of nanocarrier-based therapeutic agents to enhance the efficacy of nose-to-brain delivery systems.

## 1. Introduction

The burden caused by neurological disorders is escalating globally [1,2]. Treating brain diseases is a global challenge because there are serious limitations, such as the inability of drugs to pass the blood–brain barrier (BBB) and serious side effects [3,4]. The ability of treatments to pass through the blood–brain barrier is a major challenge for the treatment of psychiatric disorders and central nervous system (CNS) diseases [4]. The BBB’s function is to protect the CNS from xenophobic attacks from the bloodstream. However, due to its makeup, it is a stumbling block for the delivery of drugs to the CNS [3]. Moreover, some drugs have a highly hydrophilic nature and high molecular weights, leading to their low membrane permeability, and this makes it difficult for them to pass the biological barriers [5]. These limitations also affect the development of novel and effective drugs for CNS diseases, such as brain cancer, stroke, epilepsy, and traumatic brain injury [3,4,5]. Hence, the discovery of effective treatment transport to the brain is an interesting area of research.

The intrathecal administration approach is reported to have the capability to bypass the BBB. However, this strategy is reported to be painful, invasive, and have side effects [6]. Hence, the nose-to-brain delivery system is reported to be among the promising strategies for delivering brain-targeting drugs since it can bypass the BBB [7,8].

This approach is human-friendly because it is non-invasive, painless, and direct [9]. However, since it commonly uses trigeminal and olfactory epithelium nerve pathways, this administration route efficiency is limited by the small doses passing the BBB, resulting in poor drug bioavailability and weak therapeutic effects on the brain [7,10]. For instance, a drug can bypass the BBB via the olfactory epithelium, but the olfactory epithelium has a small surface area (5 cm^2^) and can only allow 25–200 µL volume to pass through, while the nasal cavity has a volume of 6 cm^3^, resulting in more drugs being absorbed by the respiratory epithelium and leading to poor drug bioavailability [10]. Hence, the improvement of this approach is a pressing need. Thus, the use of nanomedicine, including nanocarriers, has been an area of research to improve the efficacy of the nose-to-brain delivery route [7,9].

**Figure 1:**
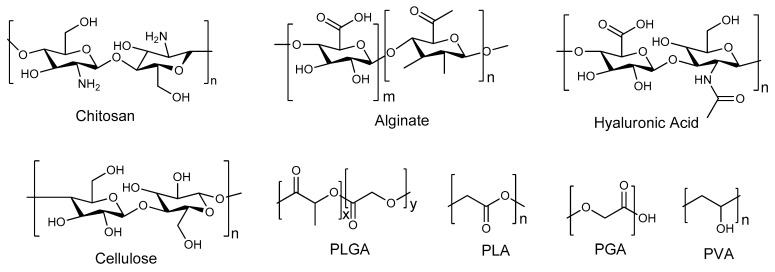
Molecular structures of polymers.

Nanocarriers are a set of therapeutic transporters with a particle size between 1 and 100 nm in diameter [6]. Polymers, both natural and synthetic, have been explored as significant constituents in the formulation of nanocarriers for biomedical applications. Examples of natural polymers that can be used in nanoparticle formulations include chitosan, alginate, hyaluronic acid, cellulose, gellan gum, and others, while synthetic polymers include poly(lactic-co-glycolic acid) (PLGA), Poly(L-lysine) (PLL), Polyethylene glycol (PEG), polyvinyl alcohol (PVA), Polylactic acid (PLA), etc. (Figure 1) [11,12]. Polymer-based nanomaterials are promising transport methods for pharmaceuticals due to their advantages, such as reduced toxicity, improved drug release, enhanced cell specificity in the brain due to surface modification by polymers, increased permeability that promotes drug delivery across the nasal epithelium and BBB, improved mucoadhesion that can prolong time of residence in the nasal cavity, and improved efficiency [13,14]. In addition, they protect the drugs from enzymatic degradation, resulting in enhanced bioavailability for brain targeting; hence, they are an ideal transport to pass through the small-surface-area pathways of the BBB [6,15]. Thus, this approach has been applied by several researchers to improve nose-to-brain drug delivery [6,16,17]. Nanotechnology-based drug delivery systems have demonstrated significant efficacy in addressing the complexities associated with nose-to-brain drug delivery [18]. These systems facilitate the targeted accumulation of drugs within the brain while substantially reducing the potential side effects associated with systemic distribution [18]. Polymer-based nanocarrier systems can also protect the encapsulated drugs from degradation and enzymatic activity while passing some barriers via nose-to-brain delivery [19]. Hence, this review focuses on new developments of polymeric nanocarriers for nose-to-brain delivery. These polymer-based nanomaterials can be fabricated in several forms, such as micelles, dendrimers, polymer–drug conjugates, nanogels, nanoliposomes, etc.

## 2. Blood–Brain Barrier (BBB)

The BBB is a selectively permeable membrane that separates the bloodstream from the interstitial fluid of the brain, enabling cerebral blood vessels to control the passage of molecules and ions between the blood and the brain [20]. The blood–brain barrier is crucial in regulating the movement of essential biological substances that support the brain’s metabolic activities and neuronal functions. Therefore, maintaining its structural and functional integrity is vital for preserving the brain’s microenvironment and ensuring its homeostasis. This dynamic separation helps maintain the stability of the neuronal microenvironment, protecting it from the fluctuating concentrations of solutes and toxins found in the circulating blood [21]. The structure of the blood–brain barrier is intricate, consisting of several key components such as specialized blood microvascular endothelium, neurons, astrocytes, and pericytes [22]. Hence, the transfer of essential nutrients to the brain and waste products from the brain to circulating blood is tightly regulated and facilitated by a large surface area and specialized transport systems. The area that includes olfactory nerve cells directly connects to the brain and cerebrospinal fluid (CSF) by bypassing the BBB [23].

The BBB consists mainly of a monolayer that is made up of tightly connected endothelial capillary cells, which permit the selective entrance of hormones and nutrients and impede the entry of toxins, pathogens, and other foreign substances (e.g., drugs) [24]. The existence of the BBB makes the pharmacological treatment of CNS disorders more challenging, as most chemical drugs and biopharmaceuticals are hindered in accessing the brain. Inadequate transportation of drugs to the brain results in reduced effectiveness of treatment and increased side effects due to buildup in other organs and tissues [25]. Bioactive agents have to cross the BBB first to reach the CNS after the systemic or oral administration of drugs. Access of drugs to the brain primarily happens via passive diffusion and active transport (transcellular or paracellular) across endothelial cells. Tight junctions result in the impermeability of the BBB to the lowest-molecular-weight molecules and large molecules, permitting only the entrance of highly lipophilic and smaller substances [26]. Now, polymeric nanocarriers encapsulated with various drugs for the treatment of CNS disorders have been explored as a potential strategy that can be utilized to bypass several barriers, including the BBB, through the nose-to-brain delivery.

Polymer-based nanoparticle drug delivery systems possess the ability to promote the therapeutic agents to reach the CNS by passing the BBB because of their functional features associated with their nanometer scale (1–100 nm) and material composition. These nanoparticles can cross the BBB assisted by their reactivity, surface area, solubility, sensitivity, and strength, among other features [2]. The nose-to-brain delivery of nanocarrier-based formulations is currently the most capable alternative for transporting drugs to the CNS. The size of nanocarriers is one of the major factors that must be well-tailored in the design of nose-to-brain delivery systems to pass the BBB [27]. In addition, particle size can impact the drug loading, release, and stability, determining the in vivo distribution, toxicity, and targeting ability towards the CNS. Particle size distribution can also influence the pharmacokinetics of nanocarriers, including the circulation time, absorption, and biodistribution. It has been shown previously that functional nanoparticles can boost intranasal drug delivery by linking mucoadhesive polymers, mucus-penetrating polymer surfactants, and surface modifications with cell-penetrating peptides, and the covalent linking of biorecognition ligands targeting the olfactory region [28]. All these factors and properties play a significant role in nose-to-brain delivery by promoting nanoparticulate-based formulations to enter the CNS through the BBB. Further information regarding nose-to-brain delivery is provided in Section 3.

## 3. Nose-to-Brain Drug Delivery Approach

**Figure 2:**
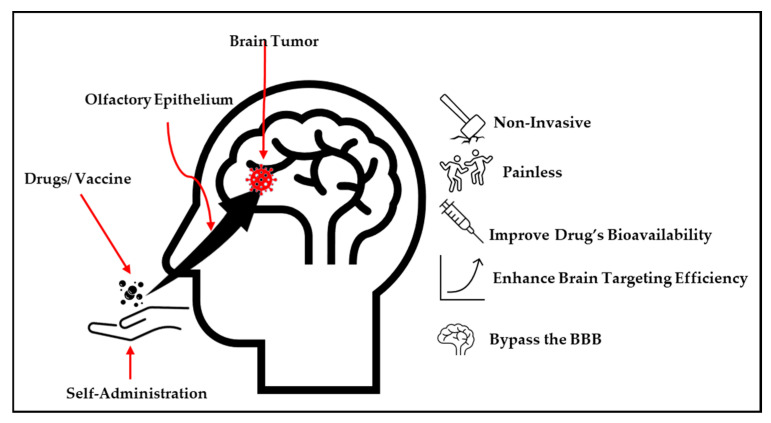
The advantages of the nose-to-brain drug delivery system.

The nose-to-brain delivery strategy involves the transport of therapeutics to the brain using the trigeminal or olfactory epithelium nerve pathways, which are situated in the upper section of the nasal cavity [29]. This strategy can bypass the blood–brain barrier, which is a stumbling block to the efficiency of therapeutic agents in treating the central nervous system and neurological diseases [22,29]. Additionally, this approach uses the only pathway that links the CNS with the external environment [29]. Notably, among the strategies employed to transport therapeutic substances into the brain, the nose-to-brain approach is showered with several advantages compared to other commonly employed strategies, as depicted in Figure 2 [5,25]. Thus, several nose-to-brain pharmaceuticals, such as nanomaterials, powders, and in situ hydrogels, have been developed [29,30].

The mechanism of the nose-to-brain delivery system involves the use of the nasal cavity regions; hence, the discussion of cellular and anatomic structure is pivotal in depicting the channels the treatment substances employ to access the brain. These include three different regions: the vestibule, respiratory, and olfactory regions [31,32,33,34]. The vestibule area is situated at the entryway of the cavity, and this region has poor drug absorption. Thus, it plays a minimal part in drug transport. The primary region of drug absorption is the respiratory region, where most of the system functions due to numerous blood vessels. This region is connected to the brain solely through the trigeminal nerve, which serves as a pathway to the brain. However, this pathway is reported to be less efficient compared to the olfactory epithelium pathway. Hence, the olfactory area (situated at the upper section of the nasal cavity) is regarded as the most important part of the nose-to-brain drug delivery system [31,32,33,34].

**Figure 3:**
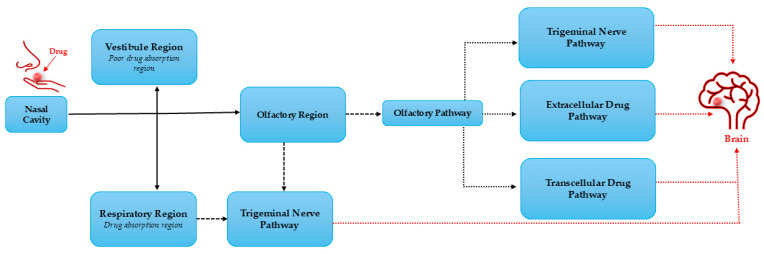
Mechanism of pathways for a drug to the brain.

As depicted in Figure 3, the olfactory region connects the outside environment with the brain. It delivers therapeutics to the brain using both the trigeminal and olfactory nerves. This pathway is regarded as the most efficient route to transport drugs to the brain. It is made up of three different channels, including the extracellular, transcellular, and intracellular pathways. The intracellular pathway involves drug transport from the olfactory region into the brain through olfactory neurons and axons, with the transcellular route transporting drugs via basal cells and the extracellular route using the channels in between the epithelium cells [31,32,33,34]. Notably, although the olfactory route delivery system is efficient, the olfactory region covers only 5–10% of the human nasal cavity, with the largest part occupied by the respiratory region. Therefore, this lower part is the site of several systematic biological events such as enzymatic drug degradation, nasal mucosa permeation, and mucociliary clearance, resulting in poor drug bioavailability to the brain [5,15,29,35]. Hence, effective strategies to enhance the efficacy of using this route are paramount. Thus, several reports depicting the use of NPs in improving the effectiveness of the nose-to-brain administration route have been documented.

## 4. Nose-to-Brain (N2B) Drug Delivery for the Treatment of Neurological Diseases

The N2B delivery approach acts primarily as a substitute for oral administration, with both systemic and local impacts. Its administration provides a hopeful pathway for the delivery of drugs across the BBB, with numerous studies proposing several key potential pathways [36]. The nasal route stands out as a highly effective option for minimally invasive drug administration, as the airway mucosa boasts good permeability and facilitates efficient absorption [36,37]. This administration route displays myriad advantages such as being a simple, fast, painless, and non-invasive delivery method where self-administration and chronic administration are possible, as depicted in Figure 2. In contrast to oral and parenteral methods, the nose-to-brain route bypasses liver metabolism, enabling a lower dose for administration [38,39].

### 4.1. The Advantages of Intranasal Administration Can Be Highlighted [18,40,41,42,43]

(a) The pathway from the nose to the brain provides ways to circumvent the blood–brain barrier through neuronal transport through olfactory and trigeminal nerves. This process leads to increased bioavailability in the central nervous system, allowing for lower drug dosages and reducing the risk of peripheral toxicity.
(b) Compared to traditional oral administration, nose-to-brain delivery bypasses hepatic first-pass metabolism and avoids gastrointestinal barriers, making it an ideal option for drugs that are sensitive to acidity and enzymes. This route is particularly beneficial for biopharmaceuticals, including proteins and peptides.
(c) For acute illnesses that need immediate treatment, quick action via intranasal administration is quite attractive. Studies have shown that 125I-insulin fully distributes throughout the brain within just 30 min following nose-to-brain administration.
(d) Nose-to-brain administration presents significant advantages for clinical applications. This non-invasive method of delivering drugs directly to the brain is not only easy to administer but also improves patient compliance and opens the door for self-medication. These qualities make it particularly beneficial for individuals undergoing long-term treatments, as well as for those with gastrointestinal issues or difficulty swallowing. Overall, this route of delivery stands out as both patient-friendly and clinically important.

In contrast, the size of the nasal cavity and the area available for intake limit the amount of drug entering the brain. Thus, the effectiveness of drug formulation for delivery methods is significantly limited due to small surface areas; for instance, the olfactory region has a small surface area of only about 5–10 cm^2^. Therefore, accurately targeting this specific region is crucial for determining both the dosage and the overall effectiveness of delivering drugs to the brain. Additionally, the mucociliary clearance system is crucial in protecting the body by preventing toxins and other foreign particles from entering. However, this mechanism also leads to low drug retention after nose-to-brain administration. Thus, overcoming this mucosal barrier is a significant challenge that must be addressed for effective nasal drug delivery.

Furthermore, the animal model utilized presents challenges when transitioning from laboratory research to clinical applications. Rodents are commonly utilized as subjects in preclinical research, and there are notable differences in the olfactory epithelium between these animals and humans. For instance, laboratory rats have an olfactory epithelium that covers 50% of their nasal surface, whereas in humans, it comprises only about 10%. This disparity highlights the limitations of using rodent models to fully understand human olfactory function. Thus, the significant differences between studies may result in less favorable results in real clinical trials when compared to those seen in preclinical studies.

Moreover, many pharmaceutical substances, along with polymers and excipients, particularly organic solvents and surfactants, can potentially cause harmful effects, inflammation, or injury to the nasal lining. Therefore, it is essential to conduct thorough examinations of these substances, given that the nose serves as both an olfactory organ and a component of the respiratory system; its physiological state affects the overall quality of life. Hence, to aid in the clinical translation of nasal preparations, it becomes vital to fully capitalize on their benefits while minimizing any drawbacks [44,45,46,47].

### 4.2. Strategies to Improve Availability in Nose-to-Brain Administration

Overcoming the BBB facilitates the administration of therapeutic agents that usually find it difficult to enter the central nervous system [24]. This approach is particularly advantageous for medications that need to be administered in large quantities to achieve their therapeutic effects. By facilitating this delivery, we can significantly reduce drug dosages, leading to a decrease in the occurrence of side effects [48]. The administration of substances via the nose-to-brain route is influenced by several factors that can present different challenges. Delivering substances systemically and accessing the brain via the nose-to-brain route still presents challenges. To address these challenges, there are three primary categories of approaches: first, the application of solubilizers, which involve altering pH levels, forming complexes, and incorporating cosolvents or surfactants; second, integrating drugs into carrier nanosystems; third, modifying the drug molecules themselves, such as using salts or hydrophilic prodrugs. Overuse of cosolvents and surfactants, along with a low pH below 4, can often result in localized adverse effects, such as irritation in the nasal passages and upper respiratory tract.

In contrast, cyclodextrins and a variety of carrier nanosystems may present safer options for nose-to-brain delivery at suitably elevated concentrations, depending on the choice of excipients and their dosage. While it is possible to achieve added benefits like improved permeation, sustained delivery, or enhanced transport directly to the brain, significant optimization efforts will be necessary. On another note, hydrophilic prodrugs can be administered at high concentrations regardless of whether they are paired with a converting enzyme. This approach has been shown to facilitate a rapid conversion from prodrug to parent drug, resulting in elevated drug levels in the brain. In the end, the decision regarding the strategy will be influenced by the unique attributes of the medication and necessitate a customized approach for every situation [49,50].

## 5. Nanocarrier-Based Drug Penetration Mechanism

Among the advantages of nanocarriers, their capability to penetrate the BBB and improve drug absorption into the brain is counted [51]. They are regarded as one of the strategies for transporting therapeutics via the nose-to-brain administration route [2,51]. Nanocarriers use numerous attributes like sensitivity, reactivity of the surface area, stability, solubility, and strength, which are limitations hindering the treatments of most CNS diseases from crossing the BBB [52]. Additionally, limitations such as enzymic degradation of drugs, rapid nasal mucociliary clearance, and poor epithelial permeation hinder nose–brain delivery efficiency [53]. Therefore, numerous reports display that the introduction of nanomaterials enhances the biodistribution and pharmacokinetics of most drugs, resulting in improved specificity and bioavailability, supplementing the efficiency of the delivery system that goes from the nose to the brain [2,53,54].

**Figure 4:**
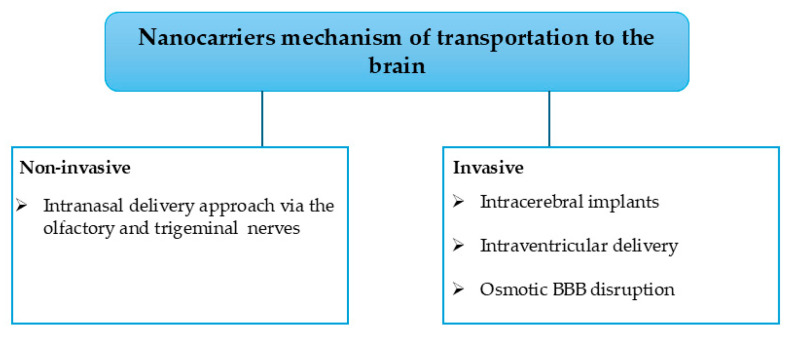
Nanocarrier strategies for brain delivery.

The strategies to deliver the nanocarriers include non-invasive and invasive approaches, as shown in Figure 4. Additionally, other alternatives can be used to transport nanocarriers [2]. The non-invasive approach involves the transportation of the nanocarriers via the transcellular pathways, whereas the invasive approach employs techniques such as focused ultrasounds, magnetic fields, and hyperosmotic solutions to alter the BBB and deliver the nanocarriers directly to the brain; however, the invasive methods are toxic in the human CNS with myriad side effects [2]. Noteworthy, the nose-to-brain route (non-invasive) uses the trigeminal and olfactory nerves to transport the NPs. However, this is hampered by the inability of larger molecules to pass through the olfactory nerve, leading to the incorporation of cell-penetrating peptide material, which leads to better efficiency with reduced cellular toxicity. Moreover, there is still ongoing research on the alternative transport of nanocarriers into the brain. Among the ongoing research methods, the iontophoresis approach, which is the use of an externally applied electric current to transport ionized molecules across the BBB, has been reported [2].

**Figure 5:**
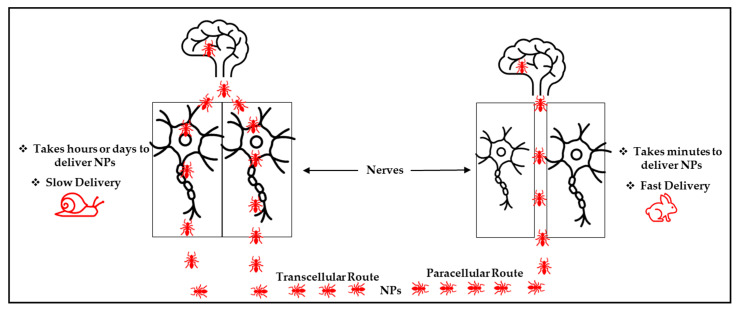
Possible mechanism of the nose-to-brain NPs delivery system.

Specifically, for the nose-to-brain NP delivery route, the olfactory nerve pathway has been a commonly used and researched route [6,55]. Thus, two possible NP penetration mechanisms have been reported for this route. These include transcellular and paracellular pathways in Figure 5 [55]. For the transcellular pathway, the nanocarriers are internalized into the olfactory receptor neurons and delivered to the brain via exocytosis and endocytosis, together with the olfactory neuron axons. Conversely (via the paracellular pathway), the NPs open a tight junction between the olfactory nerves after they are deposited on the olfactory epithelium and then use the opened space to enter the brain [55]. Notably, these nanocarrier delivery routes are similar. However, the speed of transportation of the NPs differs, as they take more time to be delivered when transported via the transcellular route (hours or days) compared to the paracellular route (minutes) [55]. This illustrates that the paracellular pathway could be a promising and efficient route for NP transportation.

## 6. Polymers in Drug Delivery Systems

**Figure 6:**
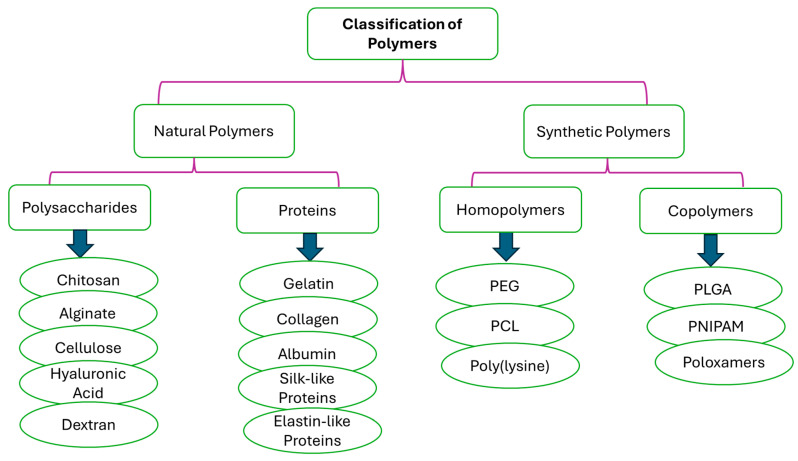
Classification of polymers based on their origin.

Polymers are the most versatile materials that have been changing the lives of people in the past decades. The selection of a polymer for the design of nanocarriers is a challenging task due to the inherent diversity of polymer structures, and it requires an in-depth understanding of the bulk and surface properties of the polymer that can provide the anticipated biological, interfacial, chemical, and mechanical functions. In addition to its physicochemical features, the choice of polymer depends on the need for extensive biochemical characterization and preclinical experiments to test its biocompatibility and safety [56]. Polymers selected for drug delivery applications are regularly categorized based on their origin as natural or synthetic polymers (Figure 6). But they can also be classified based on their water solubility (hydrophobic or hydrophilic), backbone stability (biodegradable or non-biodegradable), and chemical nature (e.g., poly (anhydride), polyester, etc.) [56]. Natural polymers, often known as biopolymers, are relatively abundant and biodegradable. The main limitations of natural polymers from a drug delivery point of view lie in the development of reproducible procedures of production and the necessity for numerous steps of purification due to their structural complexity. Important batch-to-batch variations happen because of their ‘‘bioformulation’’ in living organisms like bacteria, crustaceans, plants, or algae. Moreover, the use of natural polymers is also often hampered by their high immunogenicity [57]. However, natural polymers (polysaccharides and proteins) have been widely explored in the formulation of nanocarriers due to their biocompatibility and processability.

Natural-polymer-based nanocarriers display more resemblances to the extracellular matrix, which can result in minimally invasive properties [58]. Furthermore, natural polymer backbones are abundant in some groups that can be accessed for modification, including carboxyl groups, amino groups, hydroxyl groups, etc., making them easily accessible for further modifications [59,60]. Synthetic polymers are accessible in a broad diversity of compositions with readily controllable and adjustable properties. The principal disadvantage of these polymers is their poor biocompatibility, although PLGA and PEG are excellent exceptions [61]. Therefore, they are often linked with immunogenic or inflammatory reactions that often hinder their long-term use [62]. The shortcoming of non-biodegradable polymers used as carriers is that they need surgery to be removed from the body after they release the drug into the biological environment [63]. Polymeric conjugates and carriers are crucial in designing drug delivery systems. The main task of polymeric nanocarriers, either entrapped or conjugated—chemically bound with drug molecules—is the targeted delivery of drug molecules to specific biological sites of action. But the solubility of these nanocarriers in the physiological environment and the hindrance of the biological barrier frequently lead to suboptimal pharmacokinetics and bioavailability [64]. The key requirements for polymer nanocarriers to be employed for drug delivery include a lack of immunogenicity, water solubility, adequate pharmacokinetics, biological inertia, and the availability of functional groups permitting covalent conjugation of targeting moieties, drugs, or copolymerization [65].

The other key properties of polymers that should be considered for drug delivery applications include the following. (i) Molecular Weight and Dispersity: The molecular weight and dispersity of a polymer that can be used in drug delivery are the main factors for body distribution and, therefore, therapeutic outcomes. The polymer size is defined as “weight average molecular weight” (Mw) or “number average molecular weight” (Mn), and the ratio Mw/Mn is regularly known as dispersity index (DI). Natural polymers are polydisperse when they have a DI of more than 2 and molecular weights of more than 200 kDa, except if they are further treated to have lower-molecular-weight products. However, nucleic acids and proteins are exceptions with distinct physiological roles in the body [66]. The molecular weights of synthetic polymers can be easily controlled, and their narrow size distributions can be achieved depending on the polymerization mechanism. For instance, the molecular weight distribution of PEG is commonly low because of its synthesis by anionic polymerization, which limits the number of termination steps and chain transfer during reactions. Large-scale industrial synthesis aims at the production of monodisperse polymers because pharmacokinetic, immunological, and toxicological properties were found to be dependent on the DI. High-molecular-weight polymers like PVP were reported to be unable to go through the BBB [57]

(ii) Hydrophilicity and Electrical Charge: The functional group distribution on the surface of the polymer is another major factor that regulates the interactions with the surrounding environment. Thus, the surface charge of a polymer has been demonstrated to be a significant parameter for internalization into cells, hemocompatibility, cytotoxicity, body distribution, and complement activation [67]. Even though polyanionic polymers like derivatives of acrylic acid have been utilized in numerous commercially available formulations for oral application, few, such as sulfated glycosaminoglycans or poly(dicarboxylate phenoxyphosphazene), are offered for systemic administration [57]. (iii) Biodegradability and Drug Release: Biodegradation is not a predictor or prerequisite for successful application of a specific polymer in drug delivery, although it is often stated otherwise. For instance, both PEG and PLGA are highly successful polymeric materials, although PLGA is biodegradable, whereas PEG is not biodegradable [68]. The release kinetics of nanocarriers can be diverse, from short- to long-term drug delivery, anywhere from one day to one month to half a decade, depending on the formulation and type of application. Nevertheless, biodegradable polymers (like natural polymers) are the most vital materials in drug delivery applications because they do not require removal from the body after drug release [57].

## 7. Polymeric Nanocarrier-Based Drugs in the Treatment of CNS Diseases

**Figure 7:**
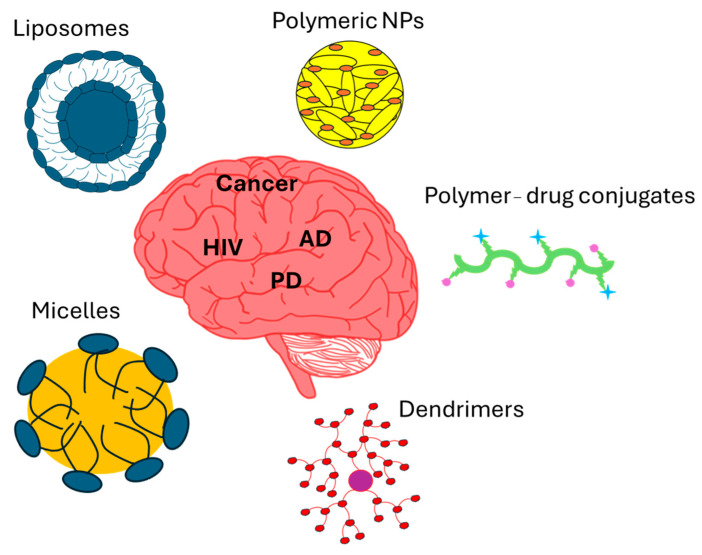
Figure showing nanocarrier-based drugs in the treatment of CNS diseases.

Recent advancements in nanocarrier technologies present exciting possibilities, particularly in areas like drug delivery, neuroprotection, and neuroregeneration. The versatility and adaptability of nanocarrier technology make it an excellent platform for creating pharmaceutical delivery systems designed for tissues. The ability to design nanoparticles that can effectively traverse the BBB while ensuring elevated drug bioavailability in neural tissue has generated considerable excitement in nanomedicine. These nanocarriers are available in different types, such as polymeric nanoparticles, liposomes, solid lipid nanoparticles, polymer–drug conjugates, dendrimers, and micelles (Figure 7). Their versatility allows for the conjugation of a range of macromolecules, such as surfactants, to achieve the desired physical or chemical properties. These nano-delivery methods offer promising new ways to treat and diagnose neurological disorders while being minimally invasive [69].

### 7.1. Cancer

A brain tumor refers to a growth or cluster of abnormal brain cells [70,71]. There are different kinds of brain tumors, which consist of both noncancerous (benign) types and malignant ones [70]. In 2024, 2,001,140 new cases and 611,720 deaths due to this disease were reported in the United States [72]. Hence, therapeutic strategies, including the fabrication of NPs, have been investigated as a potent treatment for brain tumors (Table 1). Thus, Sukumar et al. investigated the biological efficacy of a nanomaterial consisting of a combination of temozolomide, miRNAs (miR-100 and antimiR-21), and β-cyclodextrin-chitosan-modified polyfunctional gold–iron oxide nanoparticles (polyGIONs) administered via the nose-to-brain pathway to treat glioblastoma in mice [73]. They fabricated the temozolomide–polyGION loaded with miRNAs. The nanocarriers facilitated the presensitization of glioblastoma cells to the chemotherapeutic agent temozolomide that was administered systemically. Additionally, they facilitated in vivo anatomical and molecular multimodal imaging to assess the treatment effects and biodistribution of nanoparticles [73]. The fabricated nanomaterial displayed longer survival time compared to the single-entity materials (temozolomide alone or polyGIONsmiRNAs). This illustrates the importance of the NPs in enhancing the efficacy and delivery of temozolomide via the nose-to-brain route.

A nano-lipid-based in situ gel loaded with teriflunomide was created using gellan gum and Carbopol^®^ 974P, which acted as the gelling and mucoadhesive agents, respectively [74]. Interestingly, the technetium-labeled IN NLC gel formulation exhibited a Cmax in the brain that was twice as high as that of teriflunomide-loaded NLC, following both intranasal (IN) and intravenous (IV) administration. This improved absorption could be due to the combined action of the surfactant and the natural gelling polymer, which enhances the drug’s permeability. In addition, biodistribution studies show that the new formulation is rapidly delivered to the brain, suggesting it could serve as a safe and effective treatment for glioma while also lowering the risk of liver and kidney toxicity [74]. However, more clinical applications are recommended to validate the influence of the gel on the material.

Ullah et al. fabricated poly (D, L-lactic-co-glycolic) acid (PLGA)-based arginyl-glycyl-aspartic tripeptide (RGD)-conjugated paclitaxel (PTX)-loaded nanoparticles (NPs) conjugated with arginyl-glycyl-aspartic tripeptide as a nose-to-brain drug transport to treat glioblastoma [75]. Paclitaxel is associated with liver and kidney toxicity; hence, it is incorporated into the NPs to promote its specificity and mitigate its toxicity to other body organs by controlling drug release [75]. Thus, the fabricated nanomaterial displayed promising anticancer results when administered via the nose-to-brain route, displaying glioblastoma division without dividing normal cells in vivo. Additionally, the material displayed improved specificity, leading to a better anticancer effect [75]. Hence, it can be recommended as a treatment for cancer. This supplements the application of nanoparticles to improve the nose-to-brain delivery system for the treatment of CNS-related diseases.

Ferreira et al. displayed that PLGA-based nanomaterials loaded with temozolomide can be a potential treatment for glioblastoma [76]. This therapeutic nanomaterial displayed a promising anti-glioblastoma effect against several cancer cells, showing a significant tumor reduction, especially against U251 cells when administered via the nasal route, in vivo [76]. Noteworthy, the nanomaterial promotes the temozolomide nasal mucosa penetration at a controlled rate, ex vivo. In addition, the bioinspired and biomimetic nanostructure loaded with temozolomide displayed enhanced specificity, antiangiogenic effects, and tumor regression, reinforcing the importance of the nano materials in the treatment of brain tumors via the nose-to-brain delivery route. Hence, the use of NPs to improve therapeutic efficacy is a recommended strategy and a potential treatment for brain tumors. Thus, more clinical experiments were recommended [76].

Yang et al. fabricated a hyaluronan-enveloped nanomicelle material that delivers small interfering RNA to the brain through the trigeminal nerve pathway [77]. After hours of intranasal administration on GL261 tumor-bearing mice, the material invaded the brain in good concentrations, exhibiting a promising anti-glioblastoma effect as the tumor volume decreased, in vivo. Additionally, the material displayed a prolonged time of survival and no toxicity on the trigeminal nerve and nasal mucosa, and the improvement was attributed to the introduction of hyaluronic acid as its coating into the nanomaterial, which improved the viscosity of the material, increased retention time, and protected normal tissues from the drug [77]. Notably, hyaluronic acid is disruptive to the CD44 receptors that are responsible for solid tumor growth, including glioblastomas, and this could lead to tumor growth inhibition [77]. Hence, this nanomicelle was regarded as a potent treatment for glioblastoma. Kanazawa et al. developed a penetrating peptide (Tat)-modified PEG-PCL micelle material to improve anticancer efficacy [7]. The material contains stearoyl-modified bombesin mixed with Tat-conjugated polymer micelles. Following intranasal delivery in rats with orthotopic C6 glioma grafts, the nanomaterial displayed improved selective cellular uptake and tumor accumulation, resulting in good cytotoxic effects and prolonged survival time in vivo. Notably, the material was more effective when mixed compared to as a single polymeric micelle [7]. This illustrates that hybridizing nanomaterials can result in more effective therapeutic agents. Moreover, the uncoated drug resulted in weight loss after intranasal administration, whereas the drug enveloped in the nanomaterial showed no toxicity and side effects on normal tissues when administered using the same route [7]. This depicts that the introduction of the nanomicelle was pivotal in improving the efficacy of the drug when administered via the nose-to-brain route.

Ribeiro et al. developed triblock copolymer Pluronic^®^ F127-based hybrid lipid NPs as a potential treatment for glioblastoma [78]. This nanomaterial consists of perillyl alcohol entrapped into NPs. After the intranasal administration, the lipid NPs exhibited a balanced profile between mucus penetration and adhesion, resulting in enhanced mucosal retention and effective mucus diffusivity. In vitro, the nanomaterial displayed improved anti-glioblastoma activity by inhibiting tumor growth and enhancing cytotoxicity against selected glioblastoma cells (U87MG). Additionally, in vivo, the nanomaterial depicted increased translocation between the brain and nasal cavity, leading to a high concentration of perillyl acid (2.5-fold more) in relation to the unbound medication present in the brain [78]. This illustrates that the improvement in the drug delivery system was due to NPs, as they enhanced the mucus penetration ability of the drug. Hence, the strategy of loading drugs into nanomaterials is a promising approach to overcome the limitations of the nose-to-brain delivery approach.

Nanomaterial developed by Zhang et al. through encapsulation of paclitaxel with PLGA NPs exhibited good cellular uptake and improved anticancer efficacy against glioblastoma cells (U87MG cells) when administered intranasally, in vivo [79]. The material displayed a controlled drug release, attributed to the presence of PLGA. This demonstrates that PLGA could improve the specificity of the drug and improve its biodistribution, resulting in enhanced therapeutic effect without harming normal tissues. Additionally, the NPs loaded with the drug displayed high toxicity, whereas the free drug and PLGA-NPs displayed no cytotoxicity on cancer cells. Notably, it was shown that the NPs delivered via the intranasal route displayed higher efficacy compared to the same NPs transported by the intravenous route, and PLGA was responsible for the enhanced therapeutic effect [79]. This implies that the nose-to-brain approach is a promising NP delivery strategy.

Corroborating findings were reported by Chung et al. about modified PLGA NPs loaded with doxorubicin as a potent treatment for brain tumors [80]. This nanomedicine was administered to the brain through the intranasal pathway and showed an improved tumor inhibition effect when tested against C6 glioblastoma cells, inducing apoptosis while not harming normal tissues [80]. The nano-sized material showed sustained doxorubicin drug release and mucus penetration, implicating PLGA’s impact on these improvements. Moreover, incorporating RGD ligand on the NPs improved the specificity and localization of the drug to the tumor, resulting in an enhanced therapeutic effect. In contrast to the intravenous administration, the localization of the NPs improved via the intranasal administration route [80]. This indicates the good interaction between NPs and the nose-to-brain administration route in enhancing drugs’ therapeutic effect for the treatment of CNS diseases. The DSPE-PEG 2000 in situ nanogels reported by Qu et al. displayed promising anti-glioblastoma efficacy when tested on U87 and C6 cancer cells in vitro [81]. The material displayed an acceptable zeta potential and particle size as well as high-solubility properties, indicating its good efficacy for the treatment of brain tumors. It was fabricated through a combination of disulfiram and ion-sensitive nanoemulsion in situ gel, resulting in a nanomaterial with 1.2-fold and 1.6-fold longer survival times and improved tumor inhibition effect compared to its counterparts when administered via the intranasal route. Additionally, the nanogels depicted no noticeable damage to the normal cells, as confirmed by tissue-damaging studies, and loading the disulfiram into the gel protected the nasal mucosa from side effects [81]. Furthermore, the controlled release of disulfiram was also attributed to the enveloping of the drug into the gel [81]. This indicates that this kind of in situ gel can be a protector of normal tissues from xenophobic attacks. Hence, this nanomaterial is recommended for the treatment of brain tumors via the nose-to-brain route. However, more clinical studies are recommended.

The chitosan nanocapsules loaded with simvastatin reported by Bruinsmann et al. displayed anti-glioblastoma effects when tested against U-138 MG human and C6 rat glioma cells in vitro and in vivo [82]. In vitro, the nanomaterial displayed superior and comparable cytotoxic effects in comparison with non-encapsulated simvastatin against U-138 MG and C6 cell lines, respectively. Interestingly, after in vivo intranasal administration, the nanomaterial showed simvastatin bioavailability (2.4-fold more compared to the free drug) in the brain, resulting in enhanced tumor inhibition without displaying any toxicity to the brain’s normal tissues [82]. The improved drug bioavailability in the brain resulted in 78% tumor reduction, and this was attributed to chitosan due to its ability to open tight junctions in the intranasal route. Moreover, the chitosan was also responsible for the protection of the nasal mucus and other normal tissues from side effects associated with simvastatin, such as liver and kidney failure [82]. Notably, although simvastatin is not a glioblastoma agent, coating it with chitosan resulted in a glioblastoma therapeutic agent [82]. These findings converge with other reports about the enhanced therapeutic effect due to NPs in nose-to-brain delivery systems.

Ferreira et al. developed alpha-cyano-4-hydroxycinnamic acid–PGLA–chitosan NPs, conjugated them with cetuximab as a potent anti-glioblastoma agent for nose-to-brain delivery, and evaluated them for their cytotoxic effect against two glioblastoma cell lines: SW1088 and U251 [83]. The non-conjugated polymeric material displayed no noticeable anticancer effects against the two cancer cell lines in vitro. However, the conjugated polymeric nanomaterial displayed significant anti-glioblastoma activity when administered via nasal administration. This demonstrates that conjugating PLGA and chitosan together with cetuximab and cyano-4-hydroxycinnamic acid improved the anticancer effect. Furthermore, the conjugated nanomaterial exhibited antiangiogenic activity. This demonstrates that the conjugated substance prevents the generation of new blood vessels, leading to a decrease in tumor growth [83]. Hence, the material could be recommended for the prophylaxis of glioblastoma using the nose-to-brain delivery system. Jin et al. reported a successfully developed nose-to-brain delivery system utilizing poly(ethylene glycol)-polycaprolactone block polymeric micelles modified with the cell-penetrating peptide Tat (PEG-PCL-Tat). This advanced system demonstrates exceptional delivery of the anticancer drug camptothecin to the brain, resulting in significantly enhanced therapeutic efficacy in a brain tumor model [84]. Chung and co-workers created nanoparticles (NPs) using PLGA [80]. They modified these PLGA NPs with an RGD ligand to help deliver them specifically to tumors that express the Avβ3 protein [85]. The IN delivery of RGD-conjugated doxorubicin (DOX) in PLGA nanoparticles (RGD-DOX-NPs) effectively targets cancer cells and significantly reduces brain tumor growth. This approach outperforms both free DOX and unmodified OX nanoparticles in a C6-implanted GBM model. In fact, RGD-DOX-NP treatment effectively induces apoptosis specifically within the tumor region while leaving normal brain cells unaffected [80].

### 7.2. Human Immunodeficiency Virus (HIV)

HIV can infiltrate the central nervous system, where it establishes a lasting reservoir [86]. As a result, HIV infection is frequently linked to neurocognitive impairment and can lead to conditions such as HIV-associated dementia [85,86]. Neurological issues often serve as the first sign of symptomatic HIV infection in around 10–20% of individuals. Furthermore, about 60% of patients with advanced HIV show clear signs of neurological dysfunction as their illness progresses [87]. Because of the elevated statistical rate, researchers are striving to find methods to decrease this number (Table 1).

Dalpiaz et al. created a chitosan-based prodrug of AZT, referred to as U-AZT, using the nanoprecipitation technique and coated it with bile acid salts such as taurocholate and ursodeoxycholate. In vitro studies showed that murine macrophages had a higher uptake of taurocholate-coated particles compared to those coated with ursodeoxycholate [88]. In vivo studies showed the same effect in the subarachnoid spaces containing macrophages, which are major unreachable sites of HIV sanctuaries in the body. It was observed that the formulation with chitosan exhibited greater uptake of U-AZT in CSF [88].

Zhou et al. developed a drug delivery system utilizing PLGA/PPL/Pluronic F-68 nanoparticles to administer darunavir (DRV) through an intranasal route. This innovative approach addresses the obstacles to drug metabolic stability and improves the permeability of the blood–brain barrier [89]. In vivo experiments, particularly those employing intranasal administration of PLGA-DRV in wild-type mice, showed a notable rise in the brain-to-plasma ratio of DRV when contrasted to free DRV [89]. The results of the study highlight the promise of PLGA-DRV nanoformulations in diminishing HIV pathogenesis in macrophages while improving drug delivery to the brain. This approach offers a hopeful direction for the treatment of HIV-related neurological disorders [89]. Additionally, while the strategies reported have shown encouraging results, there is a need to implement more approaches to tackle the high number of individuals living with this disease.

### 7.3. Alzheimer’s Disease (AD)

AD is a long-lasting neurodegenerative disease that is widely prevalent and leads to progressive cognitive decline over time [90]. Approximately 6.9 million individuals aged 65 and above in the United States are affected by AD [90]. The number is expected to increase to 13.8 million by 2060 unless major medical advancements are achieved to either prevent or cure the disease. In 2021, Alzheimer’s accounted for 119,399 documented fatalities, as indicated on official death certificates [91]. The increasing prevalence of Alzheimer’s Disease (AD) has sparked an urgent search for innovative solutions to reduce the number of individuals affected by this condition. Researchers are exploring new strategies to improve the lives of those living with AD and support their families (Table 1).

Jiang et al. created and analyzed a HupA nanoemulsion (NE), along with a targeted version of HupA-NE modified with lactoferrin (Lf) for delivery through the nasal route [92]. In vivo experiments demonstrated that administering Lf-HupA-NE via the intranasal route significantly enhanced the delivery of the drug to the brain in comparison to HupA-NE, as indicated by differences in pharmacokinetic metrics [92]. The DTI of Lf-HupA-NE, measured at 3.2 ± 0.75, showed that brain targeting was highly effective. Moreover, the area under the curve for Lf-HupA-NE was significantly higher than that of HupA-NE [92]. Singh et al. developed modified thiolated chitosan nanoparticles utilizing an enhanced ionic gelation methodology. They assessed the efficacy of intranasal administration by monitoring the restoration of memory loss caused by scopolamine and by measuring acetylcholinesterase activity in the brains of Swiss albino mice [93]. The research discovered that administering galantamine through intranasal delivery using thiolated chitosan nanoparticles demonstrated considerable efficacy (*p* < 0.05) in comparison to both oral and nasal delivery of the solution. This inference was derived from pharmacodynamic research and biochemical evaluations of acetylcholinesterase activity in the brains of Swiss albino mice [93].

Nanaki et al. developed hybrid nanoparticles made from poly(l-lactic acid) (PLLA) and PLGA for the intranasal delivery of galantamine, a medication commonly applied for treating moderate to severe cases of AD [94]. PLGA hybrid nanoparticles were administered intranasally to healthy adult male Wistar rats. This approach led to successful delivery to the hippocampus, a brain area affected considerably by Alzheimer’s Disease, within just a few hours following a single administration.

Su and colleagues developed PEG-PLA nanoparticles loaded with miR132, an essential molecule for promoting neuronal survival in the brain [94]. Because of their net negative charge and limited solubility in water, unprotected miRNA molecules are very vulnerable to rapid breakdown or removal from the mucosal surface following nose-to-brain administration. This highlights the ongoing need for a carrier that guarantees safety, enhances stability, and provides specific targeting. Combining PLA and PEG creates a core–shell structure in water, improving nose-to-brain permeability while reducing mucociliary clearance. Animal studies have shown increased expression of SYN and PSD-95, as well as decreased neuronal cell apoptosis in peripheral nerves and the cerebral cortex, indicating the neuroprotective potential of PLGA nanoparticles [95].

Zhang and colleagues performed a comparison of in vitro and in vivo correlations (IVIVCs) between curcumin-loaded chitosan-coated PLGA nanoparticles administered intranasally and inclusion complexes of curcumin with hydroxypropyl-beta-cyclodextrin (HP-β-CD) [96]. The curcumin/–HP–β–CD complex showed enhanced cellular uptake and decreased cytotoxicity, along with exhibiting antioxidant effects at a 20 µM concentration in BV-2 cells, particularly when contrasted to curcumin–chitosan–PLGA nanoparticles [96]. Shamarekh et al. developed Protamine-coated PLGA nanoparticles suspended in a Carbopol gel for targeted delivery of Tacrine to the brain through intranasal administration [97]. This nanocomposite gel showed a rise in Cmax and AUC values in the brain during the 0–12 h period compared to intravenous and intranasal drug solutions. A histopathological analysis revealed no signs of harm, highlighting its potential for treating neurodegenerative diseases [97].

Chitosan NPs possess unique characteristics, including their mucoadhesive properties and inherent bioactivity. These features not only facilitate the delivery of drugs into the brain through the olfactory pathway but also position chitosan as a promising therapeutic agent for AD [98,99]. Encapsulating hydroxy-α-sanshool (HAS) in liposomes resulted in significantly better targeting efficacy than that of free HAS [98,99]. Liposomes are highly versatile carriers, capable of encapsulating therapeutics that are hydrophilic, hydrophobic, or amphipathic [98,99]. To ensure effective drug delivery for AD, it is crucial to address the challenges of limited penetration across the blood–brain barrier (BBB) and low oral bioavailability. To meet the challenges Rompicherla et al. [98] have proposed, Saini et al. integrated ferulic acid into PLGA-based solid lipid nanoparticles (SLNs), which improved their ability to cross lipophilic barriers. They also enhanced the surface of the SLNs by modifying them with chitosan [99]. The chitosan-coated solid lipid nanoparticles (SLNs) indicated a higher concentration of the drug in the brain, resulting in enhanced cognitive function and enhanced biochemical factor levels in both the cortex and hippocampus [99].

Lee et al. conducted a study to assess the in vivo pharmacodynamics of galantamine-loaded thiolated chitosan nanoparticles administered through the intranasal route [34]. Their findings emphasized the notable benefits of using intranasal galantamine–chitosan nanoparticles compared to both oral and nose-to-brain delivery methods, showcasing the therapeutic advantages of administering medication intranasally [34]. Musumeci and colleagues expanded on this idea by creating PLGA nanoparticles and NLC-based nanosystems specifically designed to capture a neutralizing monoclonal antibody aimed at the TNF-related apoptosis-inducing ligand (TRAIL) [100]. Research on pharmacokinetics and dynamics in an AD mouse model revealed a remarkable entrapment efficiency of 99% in both formulations, as validated by an ELISA test. Interestingly, administering the antibody–nanocarrier complex intranasally resulted in notably increased levels of the antibody in the brain compared to those achieved with the free anti-TRAIL antibody [100].

Geng et al. developed conjugated polymer-based thermoresponsive micelles (CPMs) that feature an effective thermoresponsive surface alongside a core designed for generating reactive oxygen species (ROS) [101]. This study highlights the capability of the CPMs to efficiently capture toxic Aβ aggregates at physiological temperatures. Consequently, the multifunctional nature of these micelles, which incorporates both capturing shells and ROS-generating cores, presents a promising approach to mitigate cytotoxicity associated with Aβ fibrillation [101]. Dhas et al. have developed curcumin-encapsulated chitosan-functionalized PLGA core/shell nanoparticles (CH@Cur-PLGA C/S NPs), which were administered via the intranasal route [102].

The NPs were fabricated and subjected to comprehensive characterization, confirming the successful encapsulation of curcumin with an entrapment efficiency of 75% and a particle size of approximately 200 nm. The release and permeation studies demonstrated a sustained release profile and enhanced permeability through the nasal mucosa. These results indicate the potential application of curcumin-loaded NPs for reducing oxidative stress in the brain, offering promise as a treatment strategy for Alzheimer’s Disease [102]. Zhang et al. used discoidal high-density lipoproteins (HDL-Disc) that mimic Aβ antibodies to promote the directional transport of Aβ from central to peripheral catabolism, demonstrating desirable safety and potential for translation [103]. The HDL-Disc assembly, called polyDisc, is made using a type of chitosan polymer. When it is administered through the nose, it reacts to the slightly acidic environment there. This causes the polyDisc to break down into a version of HDL-Disc that does not require a carrier. The chitosan in it sticks to the mucosal layer and temporarily opens the tight junctions. This action helps the HDL-Disc travel through the olfactory pathway and reach the brain [103]. Overall, this design demonstrates a proof of concept for developing Aβ antibody mimics that aim to enhance both central and peripheral Aβ clearance for AD treatment [103]. Furthermore, although the strategies currently reported have demonstrated promising outcomes in managing the disease, there is a necessity to explore and implement a broader array of approaches. This is particularly important given the significant number of individuals affected by this condition, which continues to rise.

### 7.4. Parkinson’s Disease (PD)

PD is a neurodegenerative disease that leads to the degeneration of dopaminergic neurons in the substantia nigra pars compacta (SNpc) [104,105]. This degeneration results in a deficiency of dopamine (DA), which ultimately gives rise to motor symptoms such as tremors, stiffness, and slowed movement (bradykinesia) [104,105]. In 2021, approximately 11.77 million individuals around the globe were living with PD [106]. Consequently, researchers are working diligently to reduce the high prevalence of PD. The global assessment of PD needs indicates a significant requirement for strategies designed to address these challenges effectively (Table 1).

Borge et al. suggested the idea of coating PLGA nanoparticles with chitosan and loading them with the anti-Parkinson’s drug ropinirole hydrochloride [107]. The chitosan coating imparted mucoadhesive properties to the nanoparticles, which markedly enhanced the drug’s permeation via sheep nose-to-brain mucosa. The improvement nearly tripled the permeability compared to the unmodified PLGA nanoparticles [107]. Bhattamisra and colleagues created chitosan nanoparticles designed for the intranasal provision of rotigotine, a dopamine agonist that is not an ergot, which has demonstrated encouraging possibilities in the therapy of PD [108]. De Oliveira et al. created an encapsulated conjugate featuring geraniol and ursodeoxycholic acid aimed at treating Parkinson’s Disease via intranasal delivery [109]. This approach tackled various drawbacks associated with geraniol, which has poor water solubility, can irritate mucous membranes, and has a short half-life in the bloodstream after oral consumption [109]. Administering this self-nanoemulsifying lipid (SLN) formulation intranasally resulted in a significantly higher concentration of geraniol in rats’ cerebrospinal fluid (CSF), all while avoiding any irritation to the mucosal lining. This observation underscores its ability to enhance the absorption of geraniol from the nasal cavity into the CSF in animal models [109].

Tengse et al. synthesized chitosan nanoparticles that combined L-DOPA and selegiline, achieving a mean particle size of 109.9 ± 1.30 nm, with a zeta potential of +26.36 ± 2.58 mV. The efficiency of entrapment was remarkable, with L-DOPA reaching 91.03 ± 3.02% and selegiline at 55.01 ± 1.97% [98]. The particles were incorporated into a nasal base that gels in situ using Poloxamer 407 [110]. The in vivo study conducted on Wistar rats highlighted the promising therapeutic potential of the new formulation by assessing its effectiveness in alleviating motor symptoms linked to PDs [110]. Asshar et al. developed a PEG-modified nanoemulsion to enhance the delivery of bromocriptine and glutathione, both of which are known for their poor oral bioavailability and absorption qualities, aiming to improve their effectiveness in managing PD [111]. Evaluations of cytotoxicity performed on neuro-2a cell lines indicated that it is safe for delivery through the nasal passage [111]. Behavioral studies have shown that optimized nanoemulsion significantly improves efficacy in PD models. This has been demonstrated through different experiments, such as the forced swimming test, locomotor activity assessment, catalepsy evaluation, rota-rod performance assessment, and akinesia test performed on Wistar rats [111].

Arisoy et al. explored the potential of WGA-grafted PLGA nanoparticles as a delivery system for IN administration of levodopa [112]. Khanna et al. achieved successful encapsulation of Nalbuphine (NLB) within solid lipid nanoparticles (SLNs) formulated from phosphatidylcholine [113]. The ability of NLB-SLN to target the brain was demonstrated through non-invasive scintigraphy, revealing that peak permeability occurred eight hours following intranasal administration [113]. Ahmad et al. developed L-DOPA encapsulated in PLGA and chitosan nanoparticles and conducted a comparative analysis of both delivery systems’ physicochemical and morphological properties. This study aimed to evaluate their potential effectiveness as carriers for delivering drugs from the nose to the brain [114]. In vivo studies conducted on Wistar rats showed that L-DOPA encapsulated in chitosan nanoparticles had twice the bioavailability and area under the curve (AUC) compared with the conventional L-DOPA solution [114]. Dimiou et al. created chitosan nanoparticles with a positive charge of +40.5 mV. These nanoparticles, which are 300 nm in size, can encapsulate L-DOPA [115]. The scientists assessed the dopamine concentrations in the brains of rats that received intranasal treatment with aqueous dispersions of GCPQ microparticles, using crystalline L-DOPA as a benchmark. They discovered that the peak plasma level of dopamine after administering the GCPQ-L-DOPA formulation was 17 times greater than that of the standard L-DOPA solution [115].

Wang et al. reported that a rotigotine (ROT)-loaded polymer micelle thermosensitive gel (ROT-PM-TSG) delivery system was engineered to enhance the solubility of the drug, prolong the residence time, and increase the concentration of the drug in the brain tissue [116]. The average particle size, encapsulation efficiency, and drug loading of the ROT-PMs were (88.62 ± 1.47) nm, (93.5 ± 0.79)%, and (19.9 ± 0.60)%. In comparison with the intravenous group, the distribution of ROT in the olfactory bulb, cerebrum, cerebellum, and striatum was 276.6%, 170.5%, 166.5%, and 184.4%, respectively [116]. Arisoy et al. prepared levodopa-loaded poly(lactide-co-glycolide) acid nanoparticles using a double emulsion–solvent evaporation method for nose-to-brain drug delivery [112]. Modified parameters, such as homogenization speed and the content of external and internal phases, were used to achieve superior loading efficiency. The F1-1 formulation consistently demonstrated a prolonged release period of up to 9 h. The in vivo experiments in mice, which used a 1-methyl-4-phenyl-1,2,3,6-tetrahydropyridine-induced PD model, strongly validated the efficacy of the selected nanoparticle formulation [112]. In summary, to manage the disease effectively, it is important to use a variety of strategies, which have shown promising results.

**Table 1:** Overview of recent developments from 2022 to 2025 in polymeric nanoparticle-based drugs for treating CNS diseases using nose-to-brain delivery.

Types of Nanocarriers	Final Findings	Size (nm), DI and Zeta (mV)	Years and References
Poly(D,L-lactic-co-glycolic) acid with temozolomide	Recommended as potential treatment for brain tumors	260 ± 60 nm, −13 ± 1, and 0.29 ± 0.05 mV	2025 [76]
Cell-penetrating peptide DP7-C with hyaluronic acid and nanomicelle	Promising intranasal delivery system for siRNAs in glioma therapy	37.84 nm, 68.06 nm, and 51.5 mV	2022 [77]
Lipid Nps with paclitaxel and transferine	Showed promising anti-glioblastoma effect	364 ± 5 nm and −43 ± 9 mV	2025 [78]
Poly(ethyleneglycol)-poly(ε-caprolactone)-copolymer modified with Tat peptide	Anticancer drug camptothecin demonstrated exceptional delivery to brain, significantly enhancing therapeutic efficacy in brain tumor model	72.6 ± 17.4 nm 5.98 ± 1.32 mV	2025 [84]
Arginylglycylaspartic acid–Doxorubicin–Poly(lactic-co-glycolic acid	Effectively reduced brain tumor growth without impacting healthy brain cells	180–200 nm	2022 [84]
Poly lactic-co-glycolic acid nanoparticles with darunavir	Diminished HIV pathogenesis in macrophages and improved drug delivery	175.1 ± 3.30 nm and −0.283 ± 0.037 mV	2024 [89]
Polymer-based thermoresponsive micelles	Effectively addressed and significantly reduced cytotoxicity linked to Aβ fibrillation	_	2022 [101]
Curcumin-encapsulated chitosan-functionalized PLGA core/shell nanoparticles	Curcumin-loaded nanoparticles may reduce brain oxidative stress, suggesting potential as Alzheimer’s treatment	200 nm	2024 [102]
Discoidal high-density lipoproteins assembly with chitosan polymer	Boosting both central and peripheral Aβ clearance is essential for effective Alzheimer’s Disease treatment	_	2023 [103]
Levodopa with Poly(lactic-co-glycolic) acid and chitosan nanoparticles	Improved bioavailability	553 ± 52 nm, 0.522, and +46.2 ± 2.3 mV	2022 [114]
Polymer micelles in thermosensitive gel	In vivo studies indicated that mean residence time of polymeric micelles and gel following nasal administration extended by 1.43 and 1.79 times compared to intravenous group	88.62 ± 1.47 nm	2023 [116]
Levodopa-loaded poly(lactide-co-glycolide) acid nanoparticles	Nanoparticle formulation consistently showed prolonged release period of up to 9 h, strongly validating its efficacy	329 ± 188.3, 0.384 ± 0.113, and −4.47 ± 0.576	2023 [112]

## 8. Parameters of Nanoparticle-Based Drugs for Intranasal Drug Delivery

Several parameters must be considered during the design and formulation of nanocarriers for intranasal drug delivery. Those parameters include particle size, zeta potential (surface charge), and surface modification. The appropriate optimization of these properties can result in effective intranasal drug delivery and potential therapeutic outcomes of nanocarriers in CNS diseases. The particle size of nanocarrier-based formulations is an essential factor in the field of drug delivery. In the context of nose-to-brain drug delivery, it is paramount to consider that smaller nanoparticles might experience minor resistance to mucous infiltration and migration along the absorption path, leading to more prospects for improved intranasal drug delivery. Larger nanoparticles might be caught by the nasal mucosa and hinder further migration to the CNS. It has been demonstrated that the entrapment of nanocarriers by the mucosa is normally influenced by a size-dependent phenomenon, whereby smaller nanoparticles (around 100 nm) penetrate significantly deeper into the epithelial layers than bigger nanocarriers (approximately 200 nm) [117,118]. Nevertheless, it is very important in the future to further elucidate the significance of particle size in intranasal drug delivery, employing more accurate imaging and quantitative techniques [119]. Gadhave et al. reported teriflunomide-loaded nano-lipid-based carbopol nanogel (TNLC) and teriflunomide-loaded nano-lipid-based carbopol gellan gum nanogel (TNLCGHG) formulations that displayed globule sizes of 109.22 ± 1.58 nm and 117.80 ± 1.75 nm, respectively, revealing that these nanocarriers can be rapidly taken up via the intranasal route [74]. Moreover, Jiang and co-workers demonstrated that the mean globule sizes of the blank NE, HupA-NE, and Lf-HupA-NE were 14.26 ± 0.16 nm, 15.24 ± 0.67 nm, and 16.78 ± 0.4 nm, respectively, which can result in even more rapid uptake during nose-to-brain drug delivery [92].

The surface charge of nanoparticles is another parameter that must be studied, knowing that the nasal mucosa is normally negatively charged. Positively charged nanoparticles are more ideal in intranasal drug delivery due to their ability to interact with the mucosa through electrostatic forces. This interaction can significantly result in bioadhesion and eventually prolong the residence time. The polymers that are suitable for the preparation of nanoparticles with a positively charged surface for nasal application are chitosan and its derivatives due to the occurrence of positive charges under the physiological conditions of the nasal environment [120,121,122]. Therefore, chitosan can be employed in the fabrication of nanoparticles to give them a positive zeta potential. A certain study focused on the nasal behavior of nanoparticle formulations demonstrated that chitosan-based nanoparticles resulted in the highest tissue interaction compared to P80-based nanoparticles, with the bound nanoparticles mainly residing in the mucosa [118]. Ex vivo mucoadhesion experiments on SLNs showed the detachment force, revealing nasal mucoadhesive strength that increased from 6.88 N (for plain SLNs) to 8.55 N (for chitosan-coated SLNs). Chitosan-coated SLNs possess a positive surface charge that promotes their interaction with the anionic mucosal membranes. Moreover, it has been demonstrated that mucoadhesion can decrease drug loss because of mucociliary clearance, leading to enhanced drug availability at the site of the absorptive surface for prolonged periods [99]. The intranasal application of mucoadhesive nanocarriers causes them to swell when they are in contact with nasal mucosal secretions and stick to the epithelial mucosa, therefore eventually resulting in their increased residence time in the nasal cavity [123].

It has been demonstrated that PLL can cause a mild positive charge since it is a cationic polymer as well, making PLGA (anionic) nanocarriers less negative, which leads to significantly higher cellular uptake, controlled release of drugs, and biodegradation during intranasal drug delivery [124]. Zhou and co-workers reported that pristine PLGA/PPL/Pluronic F-68 nanoparticles showed a significantly positive surface charge of 0.065 ± 0.195 mV, but this was subsequently changed to a negative zeta potential of 0.283 ± 0.037 mV due to the encapsulation of an anionic drug (DRV) [89]. These PLGA/PPL nanoparticles could be more suitable for the drug delivery of cationic therapeutic molecules via the nose-to-brain route. Moreover, positively charged chitosan-based nanocarriers can result in a controlled drug release mechanism in intranasal drug delivery applications. For instance, the study of spherical chitosan-polymerized HDL-Disc assembly (polyDisc) by Zhang and co-workers at PBS of pH 6.5 that mimics the nasal cavity exhibited the gradual depolymerization of spherical polyDiscs into discoidal depolyDiscs. PolyDiscs almost stripped the entire polymer after 2 h and freed the carrier, absent depolyDiscs, which had a similar size distribution as native discoidal HDL. At pH 6.5, the polyDiscs rapidly depolymerized and released approximately 60% of the HDL-Discs in 5 min, and more than 96% within 2 h. Rapid HDL-Disc release can efficiently cross the nasal epithelial cell barrier within the opening time window stimulated by the polymer of CP [103].

Surface modification of nanocarriers can play a vital role in intranasal drug delivery because these nanocarriers might lack the driving force to migrate the long distance from the nose to the brain. The lack of efficient intracellular delivery of therapeutic agents in the brain is another issue that can be addressed by surface modification of polymeric nanocarriers [125]. Molecules such as ligands (e.g., cell-penetrating peptides, Bombesin, penetratin, or Tat) are a potential approach in surface modification to improve the nose-to-brain translocation of nanoparticles [126]. The in vitro studies of Tat-modified chitosan nanoparticles using olfactory cell monolayers exhibited excellent transport potential of approximately 46%, indicating their ability to enhance intranasal drug delivery via the olfactory [127]. Bombesin (Bom) is a 14-amino-acid linear peptide, primarily obtained from frog skin, which specifically interacts with the gastrin-releasing peptide receptor (GRPR), a seven-transmembrane receptor expressed more abundantly on the membranes of tumor cells in lung, colon, breast, and brain (glioblastoma) cancers than normal tissues [124,128]. Kanazawa et al. studied the in vivo intracerebral distribution of Bom/PEG-PCL-Tat and PEG-PCL-Tat micelles loaded with coumarin, utilizing nasal administration in an orthotopic graft rat model possessing C6 glioma cells. Rats administered with Bom/PEG-PCL-Tat mixed micelles showed a clear fluorescence gradient, which was strongest in the tumor-grafted portion of the brain and less intense in normal brain tissue, whereby accumulation was not observed in rats treated with non-targeted PEG-PCL-Tat micelles. These results suggest that Bom/PEG/PCL-Tat micelles can efficiently treat glioblastoma with reduced side effects in healthy brain tissue, as they travel towards cancerous brain tissue selectively after intranasal administration [7].

**Figure 8:**
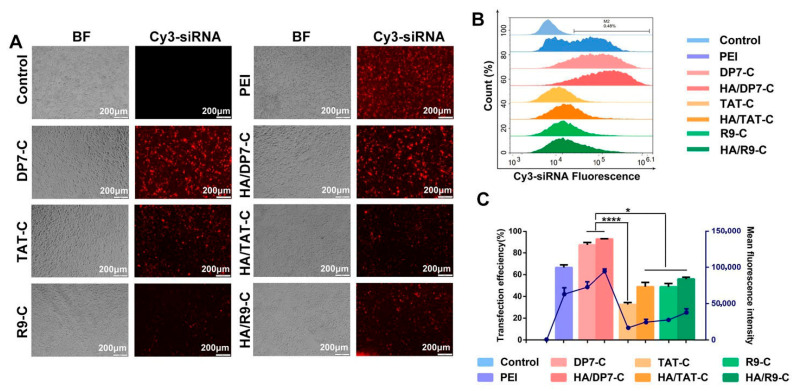
Intracellular delivery and cytotoxicity of HA/DP7-C/siRNA nanomicelles. (**A**) Fluorescence microscopy photos and (**B**) flow cytometry results of Cy3-siRNA entering GL261 cells through PEI25K, DP7-C, HA/DP7-C, TAT C, HA/TAT-C, R9-C, and HA/R9-C at 24 h. (**C**) GL261 cells were treated with HA/DP7-C/FAM-siRNA or PEI25K/FAM-siRNA for either 6 h or 24 h, followed by staining with an anti-EEA1 antibody (for early endosomes) or an anti-LAMP1 antibody (for late endosomes) along with DAPI (* *p* < 0.05 and **** *p* < 0.0001). Graphs showing the results of the cellular uptake efficiency (%) and mean fluorescence intensity. The results were obtained through a comparison with a control group. Reproduced with copyright permission from Elsevier [77].

Cell-penetrating peptides are a class of lysine- and/or arginine-rich polypeptides that are composed of less than 30 amino acids and can effectively enhance the intracellular accumulation of drugs [129]. Nanocarriers that are functionalized with cell-penetrating peptides have been demonstrated to be an auspicious approach for the delivery of siRNA, with high cellular uptake capacity and cell affinity [130]. Yang et al. evaluated whether hyaluronic acid and cell-penetrating peptide DP7-C can improve the efficiency of siRNA transfection of nanomicelles in GL261 cells. The efficiency of HA/DP7-C/siRNA and DP7-C/siRNA nanomicelles’ transfection into GL261 cells was about 92.64 ± 0.23% and 87.29 ± 2.30%, the efficiency of HA/R9-C/siRNA and R9-C/siRNA was about 55.97 ± 2.20% and 48.86 ± 5.31%, the efficiency of HA/TAT-C/siRNA and TAT-C/siRNA transfection into GL261 cells was approximately 48.86 ± 5.31% and 32.54 ± 2.42%, and the efficiency of PEI25K transfection into GL261 cells was approximately 66.50 ± 3.21% (Figure 8A–C). These results demonstrated that HA/DP7-C/siRNA nanomicelles performed better than the other two nanomicelles, HA/R9-C and cell-penetrating peptide HA/TAT-C, and the commercial polymer agent PEI25K [77]. All these parameters must be carefully considered during the design of nanoparticles for intranasal delivery because they can significantly contribute to sustained drug release, stabilization, and solubilization, and improved intranasal residence, which subsequently leads to enhanced efficacy against CNS disorders. Therefore, the perfect optimization of particle size, surface charge, and surface modification of nanoparticle–drug formulations can significantly improve the nose-to-brain delivery.

## 9. Recent Challenges and Limitations

One of the main hurdles in bringing NP-based therapies for CNS disorders into clinical practice is ensuring their long-term safety [131]. Although NPs have demonstrated significant effectiveness in preclinical studies, our understanding of how they interact with neural and systemic environments is still lacking [131]. Variations in nanoparticle size, along with differences in surface functionalization or encapsulation efficiency, can significantly impact the effectiveness of therapeutic outcomes [132]. Inorganic nanoparticles, like iron oxide and quantum dots, are under close examination due to their potential to accumulate in sensitive tissues. Their buildup can lead to oxidative stress and persistent inflammation. Biocompatible nanoparticles, such as those composed of lipids or polymers, may cause unforeseen immune responses or disrupt the carefully balanced environment of the central nervous system [133]. Conventional methods employed for small-molecule drugs or biologics frequently neglect the complex behaviors observed at the nanoscale, including interactions that depend on size, the influence of surface charge, and the dynamic alterations in biological settings. This lack of clarity in regulations can lead to significant delays in the approval of promising nanoparticle-based therapies [133].

## 10. Conclusions

The progress of the treatment of neurological diseases and CNS diseases is delayed by the lack of effective therapeutic strategies to bypass the BBB. In recent decades, biomedical researchers have been investigating the use of nanocarriers with improved nose-to-brain delivery applications. Polymers play a vital role in the formulation of nanocarriers for intranasal drug delivery applications. The therapeutic drugs are either encapsulated or conjugated with polymeric nanocarriers, with improved therapeutic outcomes towards CNS diseases. Several studies have shown that the polymeric nanocarriers with a particle size of approximately 100 nm or less achieve good penetration through the BBB and result in high cellular uptake that can significantly enhance the efficacy of their loaded drugs. Polymers such as chitosan and Carbopol in nanocarriers can act as mucoadhesive agents that can improve the absorption and permeability. These nanocarriers increase the residence time of the loaded drugs in the mucous environment, improving mucoadhesion, resulting in efficient intranasal absorption, which leads to more distribution of the drugs into the brain and improves the therapeutic effect. Among the reported polymers for the formulation of nanocarriers, PLGA is mostly highlighted as the polymer that promotes mucoadhesion, with chitosan highlighted as the better therapeutic candidate to induce penetration in tight junctions and enhance the ability of the nanomaterial to penetrate through the tight junctions of the nasal cavity, favoring a nose-to-brain delivery system.

Notably, the hybridization of polymeric nanocarriers has been reported to enhance the synergistic effect of the nanocarriers, resulting in better efficacy. However, more studies on the hybridization of these nanocarriers and their nose-to-brain administration route to treat brain diseases are a pressing need. Additionally, compared to intravenous administration, the intranasal route is a better administration route due to its ability to bypass the BBB and enhance drug concentration in the brain, and non-invasive nature. Nanocarriers are proven to improve the stability, specificity, biodistribution, and biocompatibility of therapeutic agents by controlling drug release, resulting in improved therapeutic effects and reduced side effects for CNS diseases. The incorporation of drugs into nanocarriers has been demonstrated to protect normal tissues from side effects. Moreover, polymeric nanocarrier materials administered via the intranasal route displayed better target localization compared to those administered via the intravenous route. The common trend of combining siRNAs into polymeric nanocarriers can also promote therapeutic efficacy in the treatment of brain disease via the intranasal administration route since they favor the transportation of therapeutic agents via the olfactory bulb, bypassing the BBB. Moreover, in the case of cancerous cells, siRNAs can suppress tumor growth in glioma tumors. Therefore, they have great potential for the treatment of brain tumors. However, information on nanocarriers’ interactions with neural and systemic environments and clinical trials is still lacking. Hence, further studies are recommended to enhance the progress of these polymeric nanocarriers’ therapeutic potential, leading to these therapeutic agents becoming commercially available.
